# Insight into the Quality Development and Microbial Dynamics of Meat and Meat Products

**DOI:** 10.3390/foods12091782

**Published:** 2023-04-25

**Authors:** Huiping Wang, Qian Chen, Baohua Kong

**Affiliations:** College of Food Science, Northeast Agricultural University, Harbin 150030, China; whp_1998@163.com

Meat and meat products play a vital role in the daily diet due to their desirable texture, delicious flavor and nutritional value. In order to maintain the safety and quality of meat and meat products, a comprehensive understanding of microbial composition and population, dynamic changes and microbial interactions during processing and storage is important. Recent advancements in microbial technique analysis and the roles of functional microbial strains in meat and meat products have attracted extensive attention. In this context, this Special Issue, “Meat Quality and Microbial Analysis” comprises research papers and review articles focused on the current state of knowledge on the subject.

The risk of disease caused by foodborne pathogens in meat has captured the attention of the world. Alarmingly, *Escherichia coli* O157:H7 from beef has been reported to have caused close to 46% of foodborne illnesses in 2012. Vargas et al. [1] mapped microbiological indicators at seven different locations throughout the beef processing line at four different areas on carcasses to assess microbial statistical process control parameters for each sampling point. The results showed that the sampling points for all carcass locations followed an overall trend from pre- and post-evisceration until final interventions. Collectively, the data provide microbial baselines and statistical process control parameters as a tool for supporting continuous improvements to the plant in food safety. Another foodborne pathogen, *Listeria monocytogenes*, is also widely distributed in food and related environments, possibly in planktonic and biofilm forms on food surfaces. Hu et al. [2] determined the adhesion and biofilm formation boundaries of *L. monocytogenes* ST9 under different combinations of temperature, NaCl concentration and pH and found that high temperature favored *L. monocytogenes* adhesion and biofilm formation, whereas high NaCl concentration had an inhibitory effect. In addition, sanitizers can also prevent and inhibit the growth of *L. monocytogenes*, and their concentration, temperature and duration of treatment all have significant effects on the mean log reduction in *L. monocytogenes*. Among the sanitizers, citric acid and sodium hypochlorite were the most effective against L. monocytogenes, and electrolyzed water was the least effective [3].

A new method for identifying stressed and non-stressed food-related bacteria was developed using Raman micro-spectroscopy, which achieved a classification rate of 97.6% at the genus and species levels for *E. coli* and the strain level for other food-related microbial strains [4]. Moreover, sequencing methods to observe the dynamics of microbial communities in food can detect the microbial spoilage consortium. A study based on 16S rRNA gene amplicon sequencing found that the microbial composition of pork may be similar at different storage temperatures, broadening the knowledge of microbial changes that may occur during the storage, transportation or retail of pork [5]. Qiu et al. [6] explored changes in the bacterial community during the storage of ready-to-eat chicken meat and found that pathogenic bacteria and/or spoilage bacteria directly interact, and, during storage at 4 and 22 °C, *Enterobacter* was dominant in microbial interactions, whereas *Pseudomonas* was dominant so at 22 °C. Researchers have been actively working to slow down the spoilage process of food from various aspects, such as the use of atmosphere packaging [7], the use of vacuum packaging [8], the addition of preservatives [9,10] and the addition of starter cultures [11]. However, these external treatments to slow down spoilage may lead to the simultaneous destruction of food quality characteristics and flavor, which can negatively affect consumer acceptability. De Alba et al. [12] observed the microbial quality attributes of lamb meat during the spoilage process and concluded that the mean values for the edible quality attributes of cooked meat did not change significantly during vacuum-packed storage.

Insight into the microbial community diversity of fermented meat products is essential for controlling quality development. The fermentation process is often governed by lactic acid bacteria (LAB), *Staphylococcus* and yeast. In particular, LAB are widely used in the fermentation of meat products and the fermentation systems of other raw materials. Their growth can regulate the protein degradation and oxidation in fermented meat products to a certain extent, add characteristic flavors to the products and improve the safety of fermented food [13,14]. In addition to LAB, *Staphylococcus* demonstrated excellent protein degradation ability. Wang et al. [15] purified the protease produced by *S. xylosus* A2 isolated from Harbin dry sausage, which was able to hydrolyze meat protein and obtained the hydrolysate with no cytotoxicity to HEK-293 cells. Another interesting finding was that the inoculation of some yeasts compensated for the flavor deficit of low-salt dry sausages, mainly by positively contributing to the aroma and saltiness of sausages [16,17]. However, the fermentation performance of these strains as starter cultures will vary with the change in the fermentation environment. As described by Jeong et al. [18], the number of microorganisms and various quality indexes of products at different fermentation temperatures show significant differences.

This Special Issue of “Meat Quality and Microbial Analysis” contains 18 papers covering microbial dynamics during the storage of meat and meat products; the quality of meat and meat products; microorganisms associated with fermentation, spoilage and food-borne diseases; the analysis of microorganisms in fermented meat products; and the detection and identification of microorganisms in meat and meat products.
